# Prevalence of mixed pathologies in the aging brain

**DOI:** 10.1186/s13195-014-0082-1

**Published:** 2014-11-21

**Authors:** Jasmin Rahimi, Gabor G Kovacs

**Affiliations:** Institute of Neurology, Medical University of Vienna, Waehringer Guertel 18-20, 1090 Vienna, Austria

## Abstract

The spectrum of mixed brain pathologies expands beyond accompanying vascular pathology in brains with Alzheimer’s disease-related pathology. Co-occurrence of neurodegenerative non-Alzheimer’s disease-type proteinopathies is increasingly recognized to be a frequent event in the brains of symptomatic and asymptomatic patients, particularly in older people. Owing to the evolving concept of neurodegenerative diseases, clinical and neuropathological diagnostic criteria have changed during the last decades. Autopsy-based studies differ in the selection criteria and also in the applied staining methods used. The present review summarizes the prevalence of mixed brain pathologies reported in recent community-based studies. In these cohorts, irrespective of the clinical symptoms, the frequency of Alzheimer’s disease-related pathology is between 19 and 67%, of Lewy body pathology is between 6 and 39%, of vascular pathologies is between 28 and 70%, of TDP-43 proteinopathy is between 13 and 46%, of hippocampal sclerosis is between 3 and 13% and, finally, of mixed pathologies is between 10 and 74%. Some studies also mention tauopathies. White-matter pathologies are not discussed specifically in all studies, although these lesions may be present in more than 80% of the aging brains. In summary, community-based neuropathology studies have shown that complex constellations of underlying pathologies may lead to cognitive decline, and that the number of possible combinations increases in the aging brain. These observations have implications for the prediction of the prognosis, for the development of biomarkers or therapy targets, or for the stratification of patient cohorts for genome-wide studies or, eventually, for therapy trials.

## Introduction: definition of neurodegenerative diseases and mixed pathologies

Owing to increased life expectancy, understanding the pathogenesis of age-associated cognitive decline is becoming more and more important [1]. There are many causes of dementia, but neurodegenerative diseases (NDDs) are thought to be one of the most prevalent in the aging population. Indeed, during the last century neuropathological examinations, based mostly on silver stainings, have demonstrated that the brains of the majority of the individuals with cognitive decline show Alzheimer’s disease (AD)-related pathologies, including neurofibrillary tangles and senile plaques. This observation led to the concept that AD is the most frequent NDD and cause of cognitive decline in older people.

NDDs are traditionally characterized by a selective loss of neurons in distinctive anatomical regions correlating with the predominant clinical manifestations. In addition, intracellular or extracellular deposition of misfolded proteins can be observed, resulting in the protein-based classification (proteinopathies) of these disorders [2,3]. For instance, amyloid beta (Aβ) and abnormal conformers of the prion protein are found as extracellular deposits and also in vessels in the form of cerebral amyloid angiopathy (CAA). The intracellular microtubule-associated protein tau can deposit in neurons or glial cells. Neuronal tau deposition is an important feature of frontotemporal lobar degeneration (FTLD) with Pick bodies. Progressive supranuclear palsy (PSP), corticobasal degeneration, and argyrophilic grain disease (AGD) show both neuronal and glial tau aggregates [3]. On the contrary, globular glial tauopathies show inclusions predominantly in glial cells [4]. There are further tau pathologies, including tangle-predominant dementia or various astrogliopathies, which have been described in the brains of older individuals [5,6]. Aggregates of pathologic α-synuclein are found either in the form of neuronal Lewy bodies or as Lewy neurites in disorders with Lewy bodies [2]. These comprise dementia with Lewy bodies and Parkinson’s disease. α-Synuclein-positive glial cytoplasmic inclusions (Papp-Lantos bodies) characterize multiple system atrophy. Only in recent years was Tar-DNA binding protein 43 (TDP-43), a widely expressed nuclear protein, recognized as the major protein in cases of FTLD with ubiquitin-immunoreactive inclusions with or without motor neuron disease and in sporadic motor neuron disease or amyotrophic lateral sclerosis [7]. Other relevant proteins in FTLDs are the so-called FET proteins, including the fused in sarcoma protein, Ewing’s sarcoma, and TATA-binding protein-associated factor 15 [8].

Owing to the evolving concept of NDDs, diagnostic criteria have also changed during the last decades. For instance, the Consortium to Establish a Registry for AD (CERAD) criteria were used in most of the neuropathology-based studies of AD [9]; however, these focus only on the presence of neuritic plaques (NPs). Staging for neurofibrillary tangles (NFTs) proposed by Braak, first in 1991 using silver staining and later in 2006 using immunohistochemistry for phospho-tau (AT8) [10,11], was later also recognized as an important diagnostic hallmark, and hence was reflected in the National Institute on Aging (NIA)–Reagan 1997 criteria [12]. In 2002 Thal and colleagues published that Aβ deposition also follows a hierarchical pattern in the brain [13], a concept that was implemented in the recent NIA–Alzheimer’s Association (AA) 2012 criteria [14,15]. For the neuropathological diagnosis of disorders with Lewy bodies, two different sets of criteria or staging, although partly overlapping, have been proposed. The 2003 scheme of Braak and colleagues suggested a hierarchical distribution in six stages [16]. On the contrary, the Consensus criteria on dementia with Lewy bodies distinguished three main categories. These criteria were first developed in 1996 and later revised [17-19]. In addition, cases where Lewy bodies were mainly restricted to the amygdala were included separately as an amygdala-predominant type of α-synucleinopathy [20,21].

Co-occurrence of neurodegenerative pathologies (including non-AD forms and other proteinopathies) and nondegenerative pathologies (vascular, metabolic–nutritional, and so forth) is increasingly recognized to be a frequent event in the brains of symptomatic and asymptomatic patients [5,22-24], which may be an explanation for the often complex clinical presentations. In addition, hippocampal sclerosis (HS), defined as neuronal loss and gliosis in the hippocampal formation that is out of proportion for AD-type pathology, is a frequent finding in demented patients – and is particularly associated with AD and TDP-43 pathology [25]. In sum, the term mixed or concomitant pathology can be defined as the observation of further pathological changes in addition to predominant lesions of an NDD entity, including AD or other, in the same brain [24]. In earlier studies, this term was used for the assessment of accompanying vascular pathology in brains with AD-related pathology. Later, Lewy body pathology was also considered a concomitant pathology. This definition must be expanded, however, and thus we use the term mixed pathology to indicate the concomitant presence of any neurodegenerative proteinopathy and/or further pathologic alterations, including HS, vascular lesions, or other pathologies.

Autopsy-based studies differ in the selection criteria and also in the applied staining methods and neuropathological criteria used. Comprehensive studies (that is, brain bank, community-based or other autopsy cohorts) that include the examination of different NDD-related proteins were reported only in the last few years.

The aim of the present review is to summarize and to compare the prevalence of mixed pathologies reported in recent community-based studies. The following studies are discussed in the present review (most of them are reported in several publications): Rush Memory and Aging Project (USA), Religious Orders Study (USA), Medical Research Council Cognitive Function and Ageing Study (UK), Cambridge City Over-75 s Cohort (UK), Vantaaa 85+ (Finland), Hisayama (Japan), Honolulu–Asia Aging Study (USA, Japanese–American), Adult Changes in Thought (USA), Baltimore Longitudinal Study of Ageing (USA), Oregon Brain Aging Study (USA), 90+ Study (The Leisure World Retirement Community, USA), and Vienna Trans-Danube Aging (VITA) study (Austria) (see also Table 1). These community-based studies implement more recent neuropathological diagnostic criteria or staging systems and include the evaluation of α-synucleinopathy, tau pathologies, and TDP-43. Although the definition of community- or population-based studies varies, generally a community is designated as a group of people living in a defined geographic area but being demographically and socioeconomically diverse [26].

**Table 1:** **Overview of the community-based studies discussed in the present review**

**Study**	**Country**
Rush Memory and Aging Project	USA
Religious Orders Study	USA
Medical Research Council Cognitive Function and Ageing Study	UK
Cambridge City Over-75 s Cohort	UK
Vantaa 85+	Finland
Hisayama	Japan
Honolulu–Asia Aging Study	USA
Adult Changes in Thought	USA
Baltimore Longitudinal Study of Ageing	USA
Oregon Brain Aging Study	USA
90+ Study	USA
Vienna Trans-Danube Aging study	Austria

We discuss the reasons for the variable results and we compare them with recent noncommunity-based studies, including those that implemented the proteinopathy concept in their evaluation process. Importantly, comparison of community cohorts versus clinic-based cohorts has shown that more atypical pathologies are found in the latter and hence generalization of these findings to the general population may be problematic [27].

## Differences in the methodological approach of neuropathology-based studies

The age of the participants included in the projects at baseline evaluation is crucial for the estimation of the prevalence of mixed pathologies, since younger patients tend to have only single neurodegenerative pathologies in contrast to older patients, where mixed pathologies very often contribute to their degree of cognitive decline [28,29]. A further aspect of understanding the differences in the frequency of neuropathological alterations in community-based studies is related to the fact that these use different criteria and methods (summarized in Table 2).

**Table 2:** **Summary of methodological approaches used in the community-based neuropathological studies summarized in this review**

**Study**	***n***	**Neuropathological criteria**					**Aβ**	**Tau**	**α-Syn**	**Ubi/p62**	**TDP-43**	**Vascular pathologies**	**HS**
		**BB**	**C**	**NR**	**NA**	**DLB/Br**							
MAP [27,30-33]	425	+	+	+		+^a^	+	+	+			+	+
ROS [27,30,32-35]	539	+	+	+		+^a^	+	+	+		+^b^	+	+
MRC CFAS [21,36-39]	525	+	+			+^a,c^	+	+	+	+		+	+
CC75C + [40]	224	+	+			+^a^	+	+	+	+		+	+
Vantaa 85 + ^d^ [41-43]	304	+	+			+^a^	+	+	+	+		+	+
Hisayama [44,45]	205	+		+		+^e^		+	+	+		+	
HAAS [46-48]	439	+	+			+^e^			+	+		+	+
ACT [49,50]	438	+	+	+		+^e^			+			+	+
BALS [51,52]	209	+	+			+^e^			+			+	
OBAS^d^ [53,54]	125	+	+	+		+^e^		+	+			+	+
90+ Study^d^ [55]	108	+	+			+^e^	+	+	+		+	+	+
VITA [5]	233	+	+	+	+	+^c^	+	+	+	+	+	+	+

Aβ, amyloid beta; ACT, Adult Changes in Thought; α-Syn, α-synuclein; BALS, Baltimore Longitudinal Study of Ageing; BB, Braak and Braak staging for Alzheimer’s disease; Br, Braak; C, Consortium to Establish a Registry for AD criteria; CC75C, Cambridge City Over-75 s Cohort; DLB, McKeith criteria for dementia with Lewy bodies; HAAS, Honolulu–Asia Aging Study; HS, hippocampal sclerosis; MAP, Rush Memory and Aging Project; MRC CFAS, Medical Research Council Cognitive Function and Ageing Study; *n*, number of individuals included in the studies; NA, National Institute on Aging–Alzheimer’s Association criteria; NR, National Institute on Aging–Reagan criteria; OBAS, Oregon Brain Aging Study; ROS, Religious Orders Study; TDP-43,Tar-DNA binding protein 43; Ubi, ubiquitin; VITA, Vienna Trans-Danube Aging study. ^a^DLB criteria 1996. ^b^Not assessed in all participants. ^c^Braak staging for Parkinson’s disease. ^d^Age of autopsied cohort >90 years. ^e^DLB criteria 2005.

All studies used the CERAD criteria and staging of neurofibrillary degeneration according to Braak and Braak [9,10] to assess AD-related pathology. However, only nine out of 12 studies also used immunohistochemistry for phospho-tau (Table 2). Indeed, a study by the BrainNet Europe Consortium has demonstrated that the quality of silver stainings varies considerably even in the same laboratory, which makes reproducibility and comparability of this method very difficult [56,57]. In contrast, immunohistochemistry for phospho-tau, particularly AT8, shows uniform results [56]. Moreover, immunohistochemistry is useful in detecting neuronal and glial pathologies additional to NFTs and NPs. This technique therefore facilitates the recognition of other NDDs, such as AGD, PSP, corticobasal degeneration, or less frequent tauopathies. NIA–Reagan criteria (which combine CERAD criteria and Braak and Braak staging) for the diagnosis of AD [12] have been applied in 6/12 studies, while the NIA–AA criteria [14,15] were used only in a single study (Table 2).

Depending on the date of the study and the version of the dementia with Lewy bodies Consortium diagnostic criteria [17,18], the detection of Lewy bodies varied; in particular, not all studies used immunostaining for α-synuclein (Table 2). Although amygdala-predominant Lewy body pathology frequently associates with AD [58], only two studies commented specifically on its frequency (Table 3). The importance of TDP-43-related pathology has emerged in recent years, and hence only three studies screened for this protein (Table 2). All investigators, except those from two studies [44,51], mentioned that they screened for HS. Again, definition of HS and distinction from hippocampal microinfarction is particularly important [59].

**Table 3:** **Frequency of different neuropathological variables in community-based studies**

**Study**	**Alzheimer’s disease-related pathologies**			**α-Syn**	**TDP-43**	**HS**	**Vascular pathologies**	**Mixed pathology**
	**Braak III to VI**	**CERAD**	**NIA**					
MAP [27,30]			59% (195)	15% (195)		13% (100)	46%^a^ (195)	23% (195)
ROS [27,30,35]			61% (386)	21% (386)	46% (130)	13% (100)	49%^a^ (386)	28% (386)
MRC CFAS [21,37]	52% (456)	46% (456)		39% (29% amygdala) (208)			70%^b^ (456)	
CC75C [40]	39%^c^ (213)	28% (213)		15% (213)			56%^d^ (213)	
Vantaa 85+ [41-43]	70% (304)	66% (180)	41%^e^ (180)	36% (304)		5% (132)	55%^a^ (132)	40% (132)
Hisayama^f^ [44,45]			62% (205)	29% (205)			31% (29)	10%^g^ (29)
HAAS [48]			19%^h^ (363)	10%^f^ (363)		9%^f^ (363)	28%^d^ (363)	39.5% (363)
ACT [49]	62% (438)	47% (438)		14% (438)			35%^d^ (438)	
BALS [51,52]			56%^i^ (209)	6%^f^ (34)			44%^a^ (179)	
OBAS [53]	62% (71)	44% (71)		20% (71)		7% (71)	46%^d^ (71)	
90+ Study [55]			67% (108)	6%^j^ (108)	31% (108)	29%^f^ (66)		19%^k^ (108)
VITA [5]	38% (233)	35% (233)		25% (17.2% amygdala) (233)	13% (233)	3% (233)	49%^l^ (233)	74% (233)

AD-related pathology according to CERAD was defined as moderate and frequent neuritic plaques. Using NIA–Reagan criteria, intermediate and high likelihood probabilities were included as AD-related pathology. Mixed pathologies were usually defined as AD plus any other pathology, if not further specified. Values in parentheses refer to the total number of brains autopsied and evaluated for pathologies in the referred study. ACT, Adult Changes in Thought; AD, Alzheimer’s disease; α-Syn, α-synuclein; BALS, Baltimore Longitudinal Study of Ageing; CC75C, Cambridge City Over-75 s Cohort; CERAD, Consortium to Establish a Registry for AD criteria; DLB, McKeith criteria for dementia with Lewy bodies; HAAS, Honolulu–Asia Aging Study; HS, hippocampal sclerosis; MAP, Rush Memory and Aging Project; MRC CFAS, Medical Research Council Cognitive Function and Ageing Study; NIA, National Institute on Aging; OBAS, Oregon Brain Aging Study; ROS, Religious Orders Study; TDP-43,Tar-DNA binding protein 43; VITA, Vienna Trans-Danube Aging study. ^a^Macroscopic and microscopic infarcts/brain infarcts. ^b^Any vascular disease. ^c^Severe hippocampal neurofibrillary tangles. ^d^Microinfarcts/cortical microvascular lesions. ^e^Braak stages IV to VI with moderate or frequent neuritic plaques. ^f^Data only reported for demented subjects. ^g^AD + vascular disease. ^h^Pure AD cases defined as frequent neuritic plaques according to CERAD or Braak stages V and VI. ^i^Composite AD pathology score by summing CERAD and Braak in equal measures (score >4 included). ^j^DLB high likelihood. ^k^AD + DLB/frontotemporal dementia. ^l^Vascular pathology including bleeding and ischemic lesions.

Vascular pathologies, including CAA, were evaluated in all studies. Macroscopic and microscopic lesions were identified in all studies, but some reported more extensively on the impact of these lesions and their relation to cognitive decline [52,60-64]. The evaluation and interpretation of vascular pathology in terms of cognitive decline is problematic, since there are no clear guidelines regarding assessment and relevance of these lesions [65]. The recent NIA–AA consensus guidelines provide suggestions about neuropathological characterization of vascular changes and emphasize that the number of lesions is very important [15].

## Frequency of neurodegenerative conditions in the aging brain

**Figure 1:**
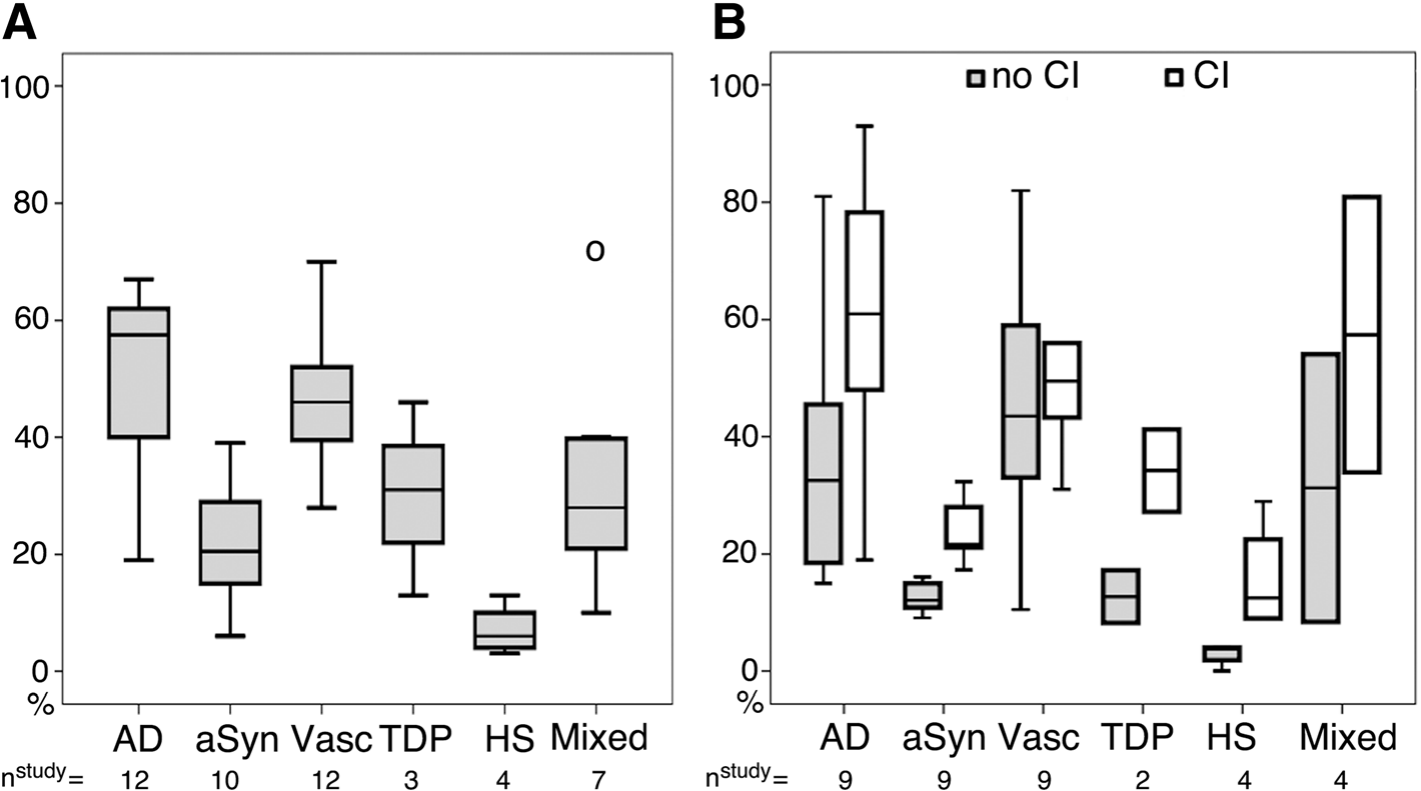
**Frequencies of different brain pathologies reported in the studies discussed in the present review.** Box-plot representation of brain pathology frequencies **(A)** for all study subjects pooled together and **(B)** separately for individuals with or without (that is, with no) cognitive impairment (CI). AD defined as the frequency of AD-related pathology starting from Braak and Braak stages III to VI or National Institute on Aging–Reagan criteria intermediate or high likelihood, ignoring other pathologies. Mixed pathologies defined as AD plus any other pathology (see also Table 2). AD, Alzheimer’s disease; aSyn, α-synuclein; HS, hippocampal sclerosis; n, number of studies that report any values; TDP, TDP-43 proteinopathy; Vasc, vascular pathology.

The frequencies of NDDs in different community-based studies are shown in Table 3 and Figure 1. AD-related pathology is the most frequent irrespective of the cognitive status of the individuals included in the study, even when moderately or highly advanced stages or scores are taken into account (that is, Braak stage ≥3; CERAD scores B and C; and NIA–Reagan and NIA–AA criteria intermediate or high likelihood) (Figure 1A). Statistical comparison of the reported values (analysis of variance, analysis of variance with Tukey’s *post hoc* test) shows that, for the studies included in the present review, the frequency of AD-related pathology (range 19 to 67%) is not significantly greater than that for vascular pathology (range 28 to 70%) or mixed pathologies (range 10 to 74%). On the contrary, only HS (range 3 to 13%) is reported as significantly less frequent than other pathologies (*P* <0.05). However, AD-related pathology mostly associates with cognitive decline (Figure 1B), supporting the notion that isocortical NFTs and NPs contribute mostly to dementia [66-68]. Findings from the Nun study identified NFT pathology as a major contributor to cognitive impairment, but the study also indicates that additional factors such as brain reserve or age contributes to the variants observed in cognitive decline [68].

The second most common neurodegenerative disorder is described as Lewy body pathology (range 6 to 39%). The evaluation of Lewy body-related pathology depends strongly on the methodology and on the regions assessed. Some studies focused on limbic and neocortical Lewy bodies; indeed, neocortical Lewy bodies are mainly associated with cognitive decline [34,66,69]. However, it has been suggested that so-called incidental Lewy body disease (brainstem) is already presymptomatic Parkinson’s disease [70], and thus the presence of Lewy bodies most probably contributes to additional symptoms and possibly also to the prognosis. Supporting this concept, evidence for a relationship between Lewy bodies in the substantia nigra and functional disability has been reported in nondemented older people [71].

Although the prevalence of HS is low in the general population (Table 3), it is twice as frequent in a demented cohort (Figure 1B) [55]. The distribution of TDP-43 pathology varies remarkably (range 13 to 46%), partly due to the fact that different antibodies are used and also due to how the presence of TDP-43 immunoreactivity is specified [35]. Furthermore, less common NDDs – such as multiple system atrophy, PSP, corticobasal degeneration, tangle-predominant dementia, FTLD-TDP [72] and even Pick bodies – are also reported in a subset of their subjects (usually below 5 to 10%) [5,27,36,41,45,51]. Application of phospho-tau immunostaining in several anatomical regions allowed the VITA study to identify a spectrum of further tau pathologies associated with the aging brain, including their association with cognitive decline [5,6]. These pathologies expand beyond the frequently detectable thorny astrocytes in the medial temporal lobe including periventricular locations [73]. Similarly to the VITA study, the brain bank-based Arizona Study of Aging and Neurodegenerative Disorders also emphasized that PSP pathology is more common in the general population than thought and that its clinical presentation varies in relation to concomitant pathologies [5,74]. All together, these observations call for caution in the interpretation of frequencies when only a few methodologies are applied (for example, silver stainings) and only restricted anatomical regions are sampled or evaluated.

## Frequency of vascular pathologies in the aging brain

Vascular pathologies are also reported as being frequent; however, due to the lack of clearly defined assessment criteria [65] there is a large range of values (Figure 1), as reported also in large autopsy cohorts [75]. Among patients with low AD-related pathology and cognitive impairment, brain infarctions were reported to be the most frequent concomitant pathology responsible for their symptoms [52,60-64,76]. The spectrum of vascular pathologies assessed at autopsy ranges from large macroscopic and smaller microscopic infarcts and lacunar infarcts, to hemorrhages, to small vessel disease and CAA. For simplification and comparability, we only present an overview of the prevalence of brain infarctions in Table 3.

Multiple lacunar infarctions were reported by the Hisayama study to be the most frequent finding in cases with dementia with a prevalence of 42% [60]. The same study reported that vascular dementia is more frequent in the Japanese population than in the western population, while the general prevalence of dementia is comparable [60]. On the contrary, the VITA study showed in the total cohort (including demented and nondemented individuals) that single micro and territorial infarcts are found frequently (up to 33%) [5]. Several studies discuss that the presence of multiple infarctions is more relevant regarding cognitive decline than the size of single infarcts [52,62,64]. While the Baltimore Longitudinal Study of Ageing suggests that only hemispheral infarcts have a strong effect, the Medical Research Council Cognitive Function and Ageing Study emphasizes that subcortical infarcts also have an influence on cognitive impairment [37,52]. The Baltimore Longitudinal Study of Ageing specifically comments on this controversy, explaining that they included subcortical infarctions also in their definition of hemispherical lesions [52]. The conclusion was that minor vascular lesions hardly contribute to cognitive decline in full-blown AD, while both mild AD-related pathology and small vessel disease interact synergistically [77]. Moreover, the lesion pattern in mixed dementia (that is, defined as AD plus vascular encephalopathy) is often associated with large infarcts, instead of with microangiopathy as in pure vascular dementia/vascular cognitive disorder [77].

Regarding the prevalence of vascular pathologies in the studies discussed here (Table 3), prevention and treatment of comorbidities such as hypertension, diabetes, alcohol consumption and previous stroke could ameliorate cognitive decline in a considerable fraction of patients [61,75,76]. Indeed, a recent population-based study also emphasized that the lack of appropriate control of risk factors for circulatory diseases combined with genetic particularities might relate to the high prevalence of vascular pathologies [78].

CAA is a frequent finding in the aging brain, being more frequent in demented people [79]. CAA is not only associated with the development of AD, but is also a highly relevant cause for hemorrhage and brain infarction [79,80]. Furthermore, CAA can be a risk factor for cognitive decline without significant AD pathology in older people [75]. The VITA study distinguished the two types of CAA as proposed by Thal and colleagues [81], and reports a significant association between higher CERAD scores, higher phase of Aβ deposition, and higher Braak stages. Furthermore, the study also found that the capillary type of CAA was related to hippocampal infarctions [5].

White-matter pathologies including periventricular and subcortical lesions are not discussed specifically in all studies. These lesions have a complex pathological basis and etiology, and may be present in more than 80% of the aging brains, somewhat (but not significantly) more in the demented [63]. Furthermore, white-matter hyperintensities, detectable on T2 and fluid-attenuated inversion recovery brain magnetic resonance imaging (MRI), are found in similar frequencies in older cohorts. Some studies suggested that these white-matter hyperintensities detected by MRI are related to CAA, but this was not confirmed in the Vantaa 85+ Study, which evaluated white-matter hyperintensities by postmortem MRI and neuropathologically assessed CAA in demented and nondemented subjects. However, this study also showed a high frequency of these alterations detectable in the postmortem MRI scans (74% in the total cohort) [82]. A longitudinal MRI study in individuals with advanced age suggested that accumulating white-matter changes in advanced age are probably driven by small-vessel ischemic disease, and even suggested there might be a link between AD pathology and white-matter integrity disruption [83].

Regarding their relevance on cognition, white-matter pathologies are controversially discussed. It has been suggested that radiologists tend to overreport periventricular and perivascular brain lesions in the MRI T2/fluid-attenuated inversion recovery compared with histologically evaluated demyelination [84]. On the contrary, routine histological assessment may underrate subcortical vascular pathology; hence, application of postmortem MRI was recommended as a complementary tool for the detection of these lesions [85].

## Frequency of mixed pathologies: high number of possible combinations

Depending on the definition of mixed pathologies – from AD plus vascular pathology to AD plus any pathology – the prevalence lies between 10 and 74%, with a higher prevalence in demented patients (Table 3 and Figure 1). Thus, although AD has been regarded as the most common cause of dementia in older people, the prevalence of mixed pathologies is on average at least as frequent. Mixed pathologies increase the odds of dementia up to almost 10 times, and up to three times compared with patients with only one pathology [86]. Moreover, the higher the Braak and Braak stage of neurofibrillary degeneration and the amount of NPs, the more probable the presence of further pathological alterations [5]. The rate of neuropathologically confirmed intermediate- and high-likelihood AD plus any other second pathology was reported as up to almost 54% in a subset of the Rush Memory and Aging Project cohort [86]. In the VITA study, where mixed pathologies were defined as any other pathologies, including also less regarded pathologies such as HS and TDP-43 proteinopathy, and non-AD tauopathies, the prevalence of mixed pathologies was over 70% [5]. Similarly to these, the Honolulu–Asia Aging Study also concluded that the co-occurrence of combined pathologies contributes to the severity of dementia and that the frequency of these pathologies increases with age [87]. The high prevalence of mixed pathologies confirmed by autopsy supports the theory that a combination of neuropathological alterations often has a cumulating effect, and – if reaching the individual’s threshold for cognitive impairment – manifests as clinical dementia [5,38].

**Figure 2:**
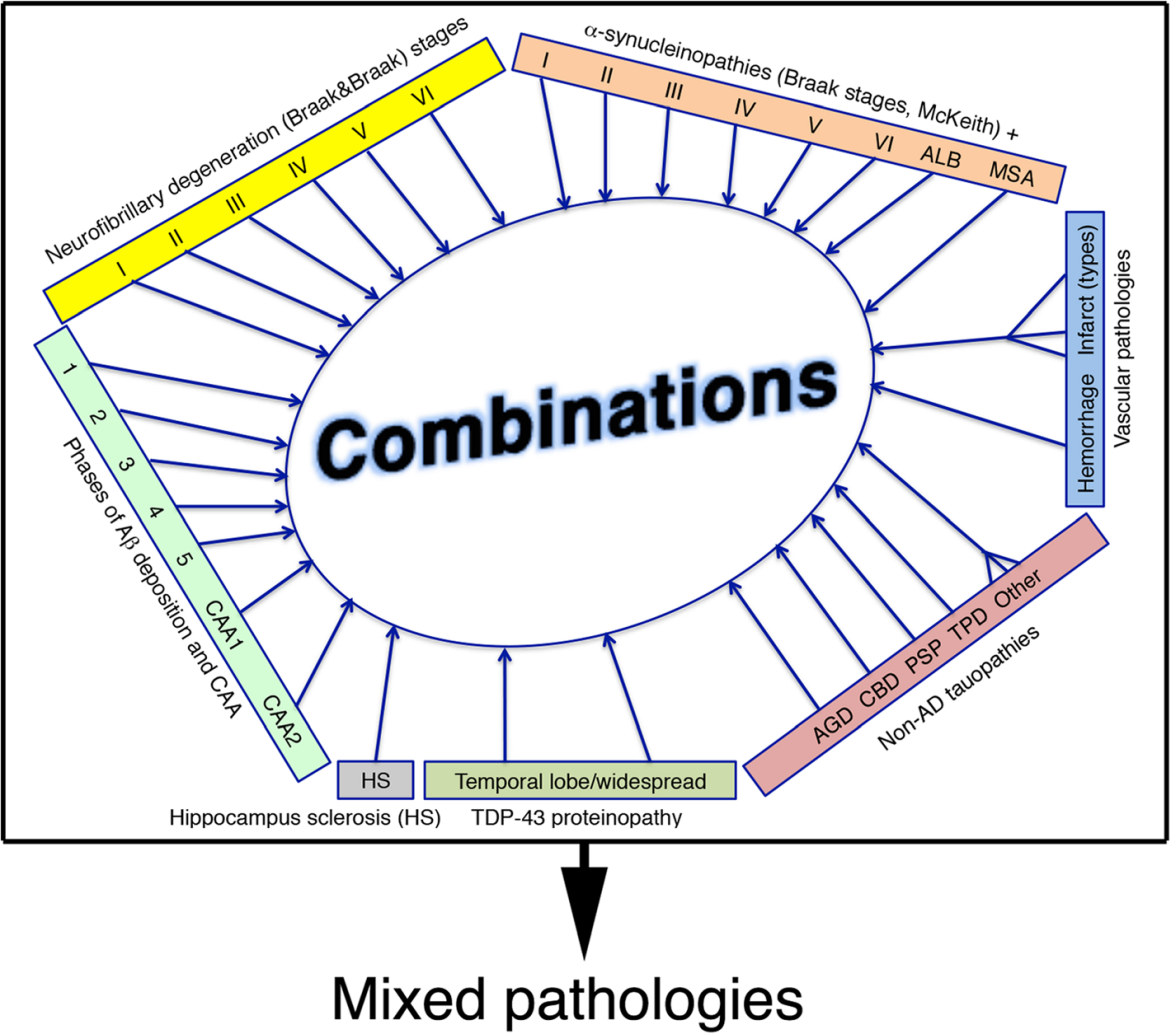
**Summary of the concept of mixed pathologies.** The holistic approach suggests that the number of combinations of different neuropathological substrates might be very high. Different combinations are covered by the umbrella term mixed pathologies. Aβ, amyloid beta; AD, Alzheimer’s disease; ALB, amygdala predominant Lewy body pathology; AGD, argyrophilic grain disease; CAA, cerebral amyloid angiopathy; CBD, corticobasal degeneration; MSA, multiple system atrophy; PSP, progressive supranuclear palsy; TDP-43, TAR DNA-binding protein 43; TPD, tangle-predominant dementia.

In addition to the studies included in the present review, further autopsy-based studies that used different recruitment and neuropathological methods also concluded that mixed pathologies are frequent and show particular increase with age [22,23,29,88]. Further studies support the concept that to understand the spectrum of pathologies in older people, non-AD type pathologies should also be evaluated in detail [25,74]. When discussing the prevalence of mixed pathologies, not only the pure frequency values are important, but also that the number of the combinations of major alterations can be very high (Figure 2) [5].

## Impact on cognitive decline

Most of the studies agree that NFTs, NPs, neocortical Lewy bodies and cerebral infarctions associate with age-related cognitive decline. We compared the frequency values of pathological variables in cases with and without cognitive impairment reported in the studies discussed in the present review using the Mann–Whitney test. This comparison shows that AD-related and Lewy body pathologies and HS are significantly *(P* <0.05) more frequent in individuals with cognitive decline. Regarding mixed and vascular pathologies and TDP-43 proteinopathy, the comparison did not reveal significant differences; however, this might also be due to differences in the definitions (that is mixed and vascular pathologies) or in the number of studies reporting on these values (that is, TDP-43 proteinopathy).

The contribution of vascular lesions to clinical dementia still remains a matter of debate. While some studies state that vascular pathologies directly contribute to dementia depending on their frequency and localization [87], others consider also a cumulative effect of this entity with co-existing NDD in the same brain [46]. Indeed, the Nun study observed that patients without lacunar infarctions seem to tolerate more AD-related pathology before presenting with dementia [89]. Furthermore, less frequent pathological changes that are highly related to dementia should not be disregarded, since they are sometimes quite challenging regarding the clinical classification of dementia [67].

The assessment of TDP-43 pathology is becoming increasingly important, since these protein aggregations are not only related to HS and FTLD-TDP, but are also associated with AD-related pathology [35]. A recent study emphasized that TDP-43 pathology is more frequent in HS compared with typical or limbic predominant AD and also shows a different distribution pattern [90]. This pathology can even expand beyond the medial temporal lobe location and be widespread, especially when associated with complex tauopathies [6]. TDP-43-related pathology and HS are two findings often observed together [25,91]. Although their frequency usually lies below 15% in the autopsy cohorts, it is noteworthy that the prevalence of HS increases above 20% in demented patients [5,55,67]. Persons with HS had lower final Mini-Mental State Examination scores [66]. HS cases were significantly older at death and showed slower rate of cognitive decline than AD subtypes [90]. Association of TDP-43 pathology with or without HS with cognitive decline or with more rapid progression of the impairment was shown in different studies [5,25,35,55,66,91]. A recent study suggests that TDP-43 is indeed a key player in the clinical features associated with AD [92]. On the contrary, one must note that some observations indicate that deposition of TDP-43 occurs in a substantial subset of cognitively normal older subjects [93].

Interestingly, an association of argyrophilic grains (tau pathology) with cognitive impairment could not be confirmed [66]. AGD can still be considered as a component that lowers the threshold for cognitive decline. On the contrary, non-AD tauopathies have an effect on the cognitive decline or may associate with further neurological symptoms leading to disability [5,74]. A combined analysis of the Rush Memory and Aging Project and the Religious Orders Study results showed that only 41% of the variance in cognitive decline can be explained by the commonly examined pathologies (AD, vascular lesions, dementia with Lewy bodies), suggesting that further causes – such as TDP-43 aggregation, HS or inflammation – should be considered in neuropathological evaluation to obtain representative explanations for cognitive alterations in aging [94].

## Conclusions and perspectives

Community-based neuropathology studies have shown that complex constellations of underlying pathologies may lead to cognitive decline, and that the number of possible combinations increases in the aging brain. However, caution is needed for the interpretation of frequency values, since the methods and criteria used and the brain regions assessed are different. Nevertheless, for clinicians these findings may be an explanation of why the diagnosis, treatment, or prediction of the prognosis can be challenging. The development of biomarkers may be a helpful tool in evaluating causes of dementia. However, one has to be aware that concomitant pathologies can bias the results of these tests. An increase of tau in the cerebrospinal fluid, for instance, can also result from disorders other than AD [28]. Furthermore, we do not know how other co-existing proteinopathies influence biomarkers and whether they can be measured via some tests in future [28]. In addition, for example, HS is clinically difficult to distinguish from AD since it not only results in memory loss but is also associated with even more severe hippocampal atrophy on MRI as seen in AD [25,30,67,91]. In addition, the complexity of disorders should be kept in mind when recruiting demented patients for genome-wide studies.

Theoretically, modifications of the most relevant proteins (Aβ, tau, α-synuclein, TDP-43) would be pivotal for evaluation simultaneously with different methods [2]. This technique should complement the detection of biomarkers associated with pathogenetic processes, and also neuroimaging and genetic analysis, in order to obtain a highly personalized diagnostic profile [2]. This concept emphasizes the continuous need for clinical–radiological–neuropathological studies to define new clusters of patients with cognitive decline, which might be useful for monitoring therapy and may open new avenues for research on pathogenesis.

Neuropathological studies should use a wide range of molecular pathological methods and should evaluate many brain regions. In addition to careful mapping of vascular lesions and histological signs of non-neurodegenerative disorders, immunostaining for p62/ubiquitin (that is, markers indicating alteration in the ubiquitin–proteasome system) may be used to screen for neurodegenerative pathology. An optimal, but less cost-effective, strategy would be to screen specifically for neurodegeneration-related proteins [3]. Strategic blocks for p62/ubiquitin immunohistochemical screening should include the hippocampus, amygdala, basal ganglia, and medulla oblongata. Screening for neurodegeneration-related proteins may include the hippocampus (that is, tau, TDP-43), the basal ganglia (that is, tau, TDP-43, Aβ), amygdala (that is, tau, TDP-43, α-synuclein), mesencephalon and medulla oblongata (that is, α-synuclein), and neocortical areas (that is, frontal, temporal for Aβ). When immunoreactivity for any protein is detected in these regions, full mapping, following diagnostic staging or classification systems is warranted. Even if the costs are higher for this strategy, omitting this concept can lead to considerable delays in the understanding of the spectrum and implications of brain pathologies in older people.

Finally, at least in the older population, targeting only single proteins for therapy might offer less success; combined preventive measures that increase the efficiency of the protein processing systems and aim to decrease vascular risk factors could be also considered.

## Note

This article is part of a series on *Cerebral multi-morbidity of the aging brain* edited by Johannes Attems and Julie Schneider. Other articles in the series can be found at http://alzres.com/series/cerebral_multimorbidity

## Abbreviations

AA: Alzheimer’s Association
AD: Alzheimer’s disease
AGD: Argyrophilic grain disease
Aβ: Amyloid beta
CAA: Cerebral amyloid angiopathy
CERAD: Consortium to establish a registry for AD
FTLD: Frontotemporal lobar degeneration
HS: Hippocampal sclerosis
MRI: Magnetic resonance imaging
NDD: Neurodegenerative disease
NFT: Neurofibrillary tangle
NIA: National Institute on Aging
NP: Neuritic plaque
PSP: Progressive supranuclear palsy
TDP-43: TAR DNA-binding protein 43
VITA: Vienna Trans-Danube Aging
